# Functional respiratory impairment and related factors in patients with interstitial pneumonia with autoimmune features (IPAF): Multicenter study from NEREA registry

**DOI:** 10.1186/s12931-023-02317-5

**Published:** 2023-01-18

**Authors:** Maria Asuncion Nieto, Olga Sanchez-Pernaute, Cristina Vadillo, Maria Jesus Rodriguez-Nieto, Fredeswinda Romero-Bueno, Belen López-Muñiz, Laura Cebrian, Maria Teresa Rio-Ramirez, Rosalia Laporta, Gema Bonilla, Tatiana Cobo, Leticia Leon, Lydia Abasolo, NEREA Group, Lydia Abasolo, Maria Asuncion Nieto, Cristina Vadillo, Irene Martín Lores, Ana Bustos Garcia de Castro, Fredeswinda Romero-Bueno, Maria Jesus Rodriguez-Nieto, Olga Sanchez Pernaute, Carmelo Palacios, Luis Gomez Carrera, Gema Bonilla, Gemma Mora Ortega, Tatiana Cobo, Belén López-Muñiz, Laura Cebrián, Hilda Godoy, Rosalia Laporta, Irene Llorente Cubas, Claudia Valenzuela, Rosario Garcia de Vicuña, Ana Jauregui, Juan Rigual, Jesús Loarce Martos, Jose Luis Morell Hita

**Affiliations:** 1 Pneumology Department, Hospital Clínico San Carlos, Madrid, Spain; 2 Universidad Complutense, Madrid, Spain; 3 Rheumatology Department, Hospital Fundación Jiménez Díaz, Madrid, Spain; 4 Rheumatology Department, Hospital Clínico San Carlos, Madrid, Spain; 5 Pneumology Department, Hospital Fundación Jiménez Díaz, Madrid, Spain; 6 Centro de Investigación Biomédica en Red de Enfermedades Respiratorias (CIBERES), Instituto de Salud Carlos III, Madrid, Spain; 7 Pneumology Department, Hospital Universitario Infanta Leonor, Madrid, Spain; 8 Rheumatology Department, Hospital Universitario Infanta Leonor, Madrid, Spain; 9 Pneumology Department, Hospital Universitario Getafe, Madrid, Spain; 10 Pneumology Department, Hospital Puerta de Hierro, Madrid, Spain; 11 Rheumatology Department, Hospital Universitario La Paz, Madrid, Spain; 12 Rheumatology Department, Hospital Universitario Infanta Sofía, Madrid, Spain; 13 Instituto de Investigacion Sanitaria San Carlos (IdISSC), Hospital Clínico San Carlos, Calle Martín Lagos, s/n. 28040, Madrid, Spain; 14 Health Sciences, Universidad Camilo Jose Cela, Madrid, Spain

**Keywords:** Interstitial pneumonia with autoimmune features, Observational study, Risk factors, Prognosis

## Abstract

**Background:**

The objective of the present study is to describe the characteristics of interstitial pneumonia with autoimmune features (IPAF) patients, to assess the incidence rate of functional respiratory impairment over time and to evaluate the influence of therapeutic alternatives on the prognosis of these patients.

**Methods:**

A longitudinal observational multicenter study was performed (NEREA registry). It was carried out by a multidisciplinary team in seven Hospitals of Madrid. Patients were included from IPAF diagnosis. Main outcome: poor prognosis as functional respiratory impairment (relative decline in FVC % defined as ≥ 5% every 6 months). Covariates: therapy, sociodemographic, clinical, radiological patterns, laboratory and functional tests. Statistics: Survival techniques were used to estimate IR per 100 patients-semester with their 95% confidence interval [CI]. The influence of covariates in prognosis were analyzed through cox multivariate regression models (hazard ratio (HR) and [CI]).

**Results:**

79 IPAF were included, with a mean and a maximum follow-up of 3.17 and 12 years respectively. Along the study, 77.2% received treatment (52 glucocorticoids, 25 mycophenolate, 21 azathioprine, 15 rituximab and 11 antifibrotics). IR was 23.9 [19.9–28.8], and 50% of IPAF developed functional respiratory impairment after 16 months from its diagnosis. Multivariate analysis: usual interstitial pneumonia (UIP) had poorer prognosis compared to non-specific interstitial pneumonia (NSIP) (p = 0.001). In NSIP, positive ANA, increased the risk of poor prognosis. In UIP, glucocorticoids (HR: 0.53 [0.34–0.83]), age (HR: 1.04 [1.01–1.07]), and Ro-antibodies (HR: 0.36 [0.19–0.65]) influenced the prognosis.

**Conclusions:**

IPAF have functional impairment during the first years of disease. Factors predicting deterioration differ between radiographic patterns. Our real-life study suggests the potential benefit of particular therapies in IPAF.

## Background

Interstitial lung disease (ILD) can be idiopathic or secondary to occupational exposures, drug toxicity and related to connective tissue diseases (CTD) [1–4]. It is essential discriminate between idiopathic and secondary ILD, because the clinical course, therapeutic strategies and prognosis among them are different [5].

CTD-ILD represents one of the most common causes of ILD [6, 7]. However, sometimes it is difficult to make a definitive diagnosis of CTD-ILD because ILD can be a sole manifestation of certain CTDs, or the symptoms suggestive of CTD may be too subtle [8–10]. In the absence of a defined CTD, 10–20% of patients with idiopathic ILD have systemic symptoms and serological abnormalities suggestive of an autoimmune process [11, 12].

To differentiate between idiopathic or ILD associated with “hidden” collagen disease [8], several definitions and nomenclatures have been suggested. The European Respiratory Society (ERS) and the American Thoracic Society (ATS) Task Force proposed the new term ‘interstitial pneumonia with autoimmune features (IPAF)’ reporting classification criteria in 2015 [13]. It has the advantage of removing previous nomenclatures, creating a framework to study a more uniform population. Up to date, several studies regarding IPAF have been developed, but most of them are short term, single center and retrospective [14, 15]. It seems that IPAF is likely to be comprised of a heterogeneous group of patients, as regards as different patient phenotypes and therapeutic targets, but findings to clarify IPAF natural history remain diverse, it is still unclear whether IPAF is a unique clinical entity or an idiopathic ILD [16], and the management of IPAF is still poorly known.

Nowadays, IPAF is considered and orphan disorder lacking a standard of care, in which drug repurposing from CTD and the use of antifibrotics is usually done in a pragmatic approach and. therapeutic decisions must be multidisciplinary t [17, 18]. Recently, a small observational study has shown the potential benefit of glucocorticoids and micofenolate mofetil on the functional progression in IPAF [19]. Regarding antifibrotics, two clinical trials including an IPAF subgroup, have showed positive outcomes [20, 21].

With the purpose of a better understanding of CTD-ILD including IPAF patients management, we have launched an observational multicenter registry (NEREA), with an interdisciplinary CTD-ILD team composed by pneumologists and rheumatologists from 7 hospitals of Madrid since 2017. The objective of the present study is to describe the characteristics of IPAF patients from NEREA, to assess the incidence rate of functional respiratory impairment over time and to evaluate the influence of therapeutic alternatives on the prognosis of these patients.

## Methods

### Setting

Comprised 7 public hospitals fom Madrid, Spain, -Hospital Clínico San Carlos (HCSC), Hospital Infanta Leonor (HiLe), Hospital Infanta Sofía (HIS), Hospital Puerta de Hierro (HPH), Hopsital Getafe (HUG), Hospital de la Paz (HULP), and Hospital Fundación Jiménez Díaz (FJD)-, covering catchments area of approximately 400,000 people each.

### Study design

It’s a multicenter observational longitudinal study, prospective since 2017. Patients were included from Feb 2007 to Dec 2019 and followed until loss of follow-up or end of study (Jan 2021).

### Population and patients

All patients were enrolled, included and followed-up at special multidisciplinary CTD-ILD-units carried out by pneumologists and rheumatologists.

Patient data were recorded in the NEREA Registy (pNEumology RhEumatology Autoimmune disease). It is a digital platform that generates an electronic case report form through the REDCap tool. Patients needed to meet any autoimmune disorder according to EULAR/ACR classification criteria [22, 23], and a diagnosis of ILD according to the ERS/ATS guidelines [6]. Patients with IPAF were also included once the diagnostic criteria from the ERS/ATS task force have been applied (retrospectively or prospectively according to the time of diagnosis) [13].

Data in this project were obtained during clinical practice, and patients provided signed informed consent. The study was conducted in accordance with the Declaration of Helsinki and Good Clinical Practices, and approved by the local IRBs (17/170_E_BS).

### Variables

The primary outcome was the presence of pulmonary functional impairment defined as a relative decline in forced vital capacity predicted (FVC%) of at least 5% per visit compared to the previous one. Pulmonary function tests (PFT) were performed at baseline and every 6 months.

The following baseline **covariates** were included: **a)** Sociodemographic characteristics; **b)** Clinical characteristics; **c)** IPAF classification criteria (Table 2); **d)** PFTs **e)** ILD radiographic patterns.

Treatments prescribed prior to IPAF diagnosis and during follow-up were also included as covariates. **f1)** Prior glucocorticoids (up to 2 months before IPAF diagnosis); and their use during follow-up. **f2)** Prior use of disease modifying antirheumatic drugs (DMARDs) defined as any DMARD administered in the 6 months prior to IPAF diagnosis; and concurrent DMARDs recorded during the study period. DMARDs included **f2a)** conventional syntetic DMARDs (csDMARDs): azathioprine (AZA), mycophenolate mofetil (MMF), methotrexate (MTX), leflunomide (LEF), calcineurin inhibitors (tacrolimus, cyclosporin) and antimalarials (AM); and **f2b)** biologic agents (targeted synthetic or biologic DMARDs (ts/bDMARDs): TNF inhibitors (Anti-TNF), abatacept, rituximab (RTX), tocilizumab (TZL), tofacinib. **f3)** Antifibrotic agents pirfenidone (PIRF) or nintedanib (NINT) during the follow-up. **f4)** Prior oxygen therapy (up to 2 months before IPAF diagnosis); and their use during follow-up. **g)** Time calendar: year of IPAF diagnosis (in five year intervals).

### Statistical analysis

Descriptive statistics were expressed as mean and standard deviation or median and interquartile rank for continuous variables, while proportions for categorical variables.

Survival techniques were used to estimate the incidence rate of functional impairment (IR), expressed per 100 patients-semester with their respective 95% confidence interval [CI]. Kaplan–Meier curves were set to account for functional impairment over time. Time of observation comprised the period from the IPAF diagnosis to loss of follow-up, main outcome or end of study. The mode in which drug prescription was done -real-life conditions- hampered the categorization of therapeutic options, being analyzed in a time-dependent manner. They were divided in periods according to the patient’s visits with their corresponding respiratory function tests that determined the presence or not of an event in that time frame. In every period, if a patient was taking any DMARD/antifibrotic more than three months was considered exposed. The exception was rituximab that had to be exposed at least 6 months. Glucocorticoids was considered exposed if the patient was on this medication at least 2 months during the period analyzed.

Cox bivariate analyses were done to assess the differences between functional impairment and covariates. Cox multivariate regression models (adjusted for centers, calendar time, age, sex, disease severity at baseline and all variables with a p value less than 0.15 in the bivariate analysis) were run to examine the influence of therapy alternatives and other covariates on functional impairment. Three multivariate models were developed: one with the entire population and the remaining two stratified by radiographic patterns. Results were expressed by hazard ratio (HR) and [CI]. We limited the number of variables in the multivariate model following the rule of Freeman and the value of 10 events per variable [24]. Missing values more than 10% were not used in the multivariate analysis. Proportional hazard assumption was tested using Schoenfeld residuals and the scaled Schoenfeld residuals. All analyses were performed using Stata v.13 statistical software (Stata Corp., College Station, TX, USA). A two-tailed *p* < 0.05 was considered statistical signification.

## Results

Seventy nine IPAF patients were included, with a total follow up of 462.8 patients-semester, a median of 3.17 ± 2.7 years and a maximum follow-up of 12.3 years. Table 1 includes a description of the patients. Many of them were women (79%) in their sixties. In 55% of them, ILD diagnosis preceded any registered autoimmune substrate (median time 8.5[28–4.9] months). Interestingly, 10% of the patients had autoimmune features previously to ILD (median time 1.7 [1–5.3] years). Diagnosis of IPAF was stablished in the rest of them at the same time ± 1 month of ILD diagnosis.

**Table 1:** Baseline sociodemographic, clinical, functional, radiologic characteristics of the patients

Variables	N = 79
Sociodemographic	
Women, n (%)	53 (79)
Mean age at ILD diagnosis ± SD, years	66.5 ± 11
Smokers (active and formers), n (%)	11 (14)
BMI, median [p25-75]	26.5 [24–31.5]
Hospital center, n (%)	
FJD	34 (43)
HCSC	23 (29)
HiLe	6 (7, 6)
HIS	2 (2, 5)
HPH	4 (5)
HUG	6 (7.6)
HULP	4 (5.1)
Comorbidity n (%) (n = 74)	
Total	51 (68.9)
Comorbidity, n (%) (n = 74)	
Hypertension	27 (36.5)
Cholesterol	22 (29.7)
Diabetes Mellitus	5 (6.7)
Ischemic heart disease	9 (12.2)
Cerebrovascular disease	4 (5.4)
Peripheral vascular disease	2 (2.7)
Hypothyroidism	9 (12.2)
Liver disease	4 (5.4)
History of cancer	7 (9.5)
COPD-Bronchiectasis	8 (10.8)
Renal failure (dialysis)	2 (2.7)
GER	4 (5.4)
History of cancer	7 (9.5)
ILD associated (HRCT), n (%)	
Fibrotic (n = 54)	23 (42.6)
Emphysema (n = 54)	10 (18.5)
PHT (n = 63)	5 (8)
PTF parameters, median [p25–75]	
FVC% (n = 79)	88 [74–101]
DLCO% (n = 68)	64 [50–81.5]
Radiographic ILD pattern (HRCT) n (%)	
UIP	29 (36.7)
NSIP	37 (46.8)
Others	13 (16.5)
Laboratory, median [p25–75]	
ESR	34 [17–50]
Therapy previous IPAF, n (%)	
Glucocorticoids -DAMRDs	26 (33)
Regimen of therapy during the follow-up, n (%)	
None	18 (22.7)
Glucocorticoids and/or DMARDs and/or Anti-Fibrotic	61 (77.2)
Glucocorticoids alone	5 (6.3)
DMARDs and/or Anti-Fibrotic	9 (11.5)
Glucocorticoids and DMARDs and/or Anti-Fibrotic	47 (59.5)

Description of the therapy used previous and along the study follow-up

Centers: Hospital Clínico San Carlos (HCSC), Hospital Infanta Leonor (HiLe), Hospital Infanta Sofía (HIS); Hospital Puerta de Hierro (HPH) Hospital Getafe (HUG), Hospital Universitario de la Paz (HULP), and Hospital Fundación Jiménez Díaz (FJD). COPD: chronic Obstructive pulmonary disease; GER: gastro esophageal reflux; ILD: interstitial lung disease; PTF: Pulmonary functional tests; PHT: pulmonary hypertension; FVC%: predicted forced vital capacity; DLCO%: predicted diffusing capacity of the lungs for carbon monoxide; UIP: usual interstitial pneumonia; NSIP: non specific interstitial pneumonia and others: indeterminate (5); lymphoid interstitial pneumonia (LIP) (1); cryptogenic organizing pneumonia (COP) (1); alveolar pattern (1), mixed pattern [NSIP-COP (2), alveolar-COP (3)]; ESR: erythrocyte sedimentation rate; DMARDs: disease modifying antirheumatic drugs

NSIP was the predominant radiological pattern (Tables 1 and 2). The mean baseline FVC% and DLCO% were 88.5 ± 22.7 and 64.2 ± 19.3 respectively (Table 1). As regards to DLCO%, baseline levels were normal (> 80%) in 27% of the patients; whereas 30% and 9% had moderate (41–60%) and severe reduction respectively (< 40%) [25].

**Table 2:** IPAF Classification criteria of patients from the study: clinical domain; serological domain and morphological domain

Clinical domain (n = 54), n (%)	
Raynaud’s phenomenon	19 (35, 8)
Arthralgia / arthritis	25 (46, 2)
Distal digital fissuring	5 (9, 3)
Unexplained digital edema	4 (7, 4)
Palmar telangiectasia	1 (1, 85)
Serological domain, n (%)	
ANA ≥ 1/320 (n = 73)	59 (80.8)
Anti-Ro (n = 72)	18 (25)
RF ≥ 40 (n = 65)	19 (29.2)
Anti-CCP (n = 50)	6 (12)
Anti-RNP (n = 69)	4 (5.7)
Anti-PM Scl (n = 62)	1 (1.6)
Morphological domain (n = 79)	
Radiologic patterns by HRCT, n (%)	
NSIP	37 (46.8)
NSIP/COP	2 (2.5)
COP	1 (1.3)
LIP	1 (1.3)
UIP	29 (36.7)
Others	9 (11.4)
Histopathological patterns by lung biopsy, n = 18 (HCSC y FJD), n	
NSIP	3
UIP	5
Interstitial lymphoid aggregates with germinal centers	5
OP	1
NSIP/OP	2
Others	2
Multi-compartment involvement, n (%)	
Unexplained pulmonary vasculopathy (n = 54)	4 (7,4)
Unexplained intrinsic airways disease (n = 54)	3 (5,5)
Unexplained pulmonary vasculopathy (n = 54)	4 (7,9)
Unexplained pericardial/pleural effusion/thickening (n = 54)	3 (5,5)

*HRCT* high-resolution computed tomography, *ANA* antinuclear antibody, *RF* rheumatoid factor. Anti-Ro: anti-Sjögren’s syndrome related antigen A; Anti-CCP: anticyclic citrullinated peptide antibodies; Anti-RNP: anti-nuclear ribonucleoprotein U1; ANTI PM Scl: polymyositis- scleroderma overlap antibody NSIP: non-specific interstitial pneumonia; COP: cryptogenic organizing pneumonia; LIP: lymphoid interstitial pneumonia

**Fig. 1:**
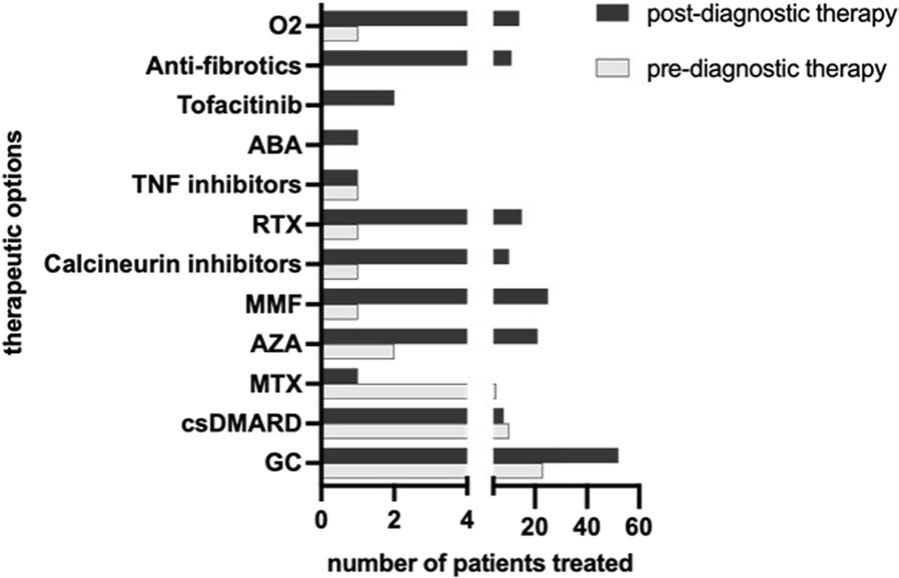
Type of drugs received in IPAF patients

Concerning medication, 33% of the patients had been previously trated with glucocorticoids and or DMARDs, mainly MTX and LEF (Table 1 and Fig. 1), due to symptoms before IPAF diagnosis, essentially arthritis. During the study period, 77.2% of the patients received treatment. Patients taking glucocorticoids increased to 66% and 48% of the patients were taking csDMARDs at some point during the follow-up (Fig. 1). Regarding biologic agents, 19% of the patients used them during the study period (mainly RTX). Antifibrotics were prescribed in 14% of the patients during the follow-up (n = 6 PIRF and n = 5 NINT). During the follow-up, the most frequent prescribed was the monotherapy regimen (84%), being AZA and MMF the most frequent (n = 41), followed by RTX (n = 10), calcineurin inhibitors (n = 7), and antifibrotics (n = 5). The other regimen was the combination: 54% of them were on DMARDs + DMARDs and 46% (n = 6) were on DMARDs + antifibrotics [RTX + antifibrotics (n = 1); MMF + antifibrotics (n = 2); AZA + Antifibrotics (n = 1); and AM + antifibrotics (n = 2)].

**Fig. 2:**
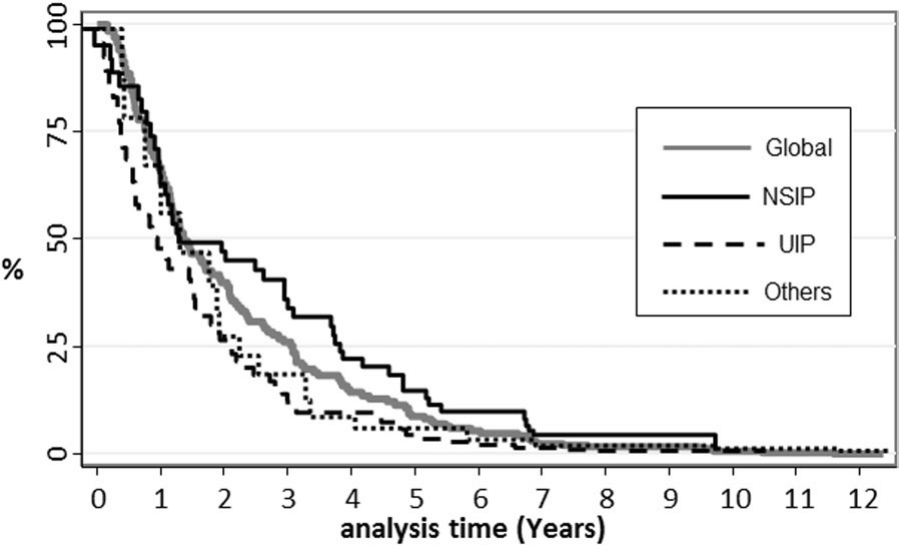
Kaplan–Meier survival estimate curve. Pulmonary functional impairment global and by radiographic patterns

Through the study, 50 patients presented 111 pulmonary impairment events over time. As a whole, 37% did not developed functional impairment, whereas 28% and 35% experimented 1 and 2 or more events of functional impairment respectively. The IR was 23.9 [19.9–28.8]. As shown in the Kaplan Meyer survival curve (Fig. 2), 33%, 50% and 60% of the patients suffered functional deterioration at 12, 16 and 24 months respectively after IPAF diagnosis.

Table 3 shows the IR of deterioration by different variables. As expected, IR was higher in UIP than in NSIP or any other pattern (see also Fig. 2). Regarding serologic domains, patients with at least medium titers of ANA antibodies had a higher IR compared to those with lower titers or a negative test. IR in patients taking glucocorticoids prior to IPAF diagnosis was similar to those without glucocorticoids. Likewise, patients on DMARDs prior to IPAF diagnosis had similar IR compared to those without. After IPAF diagnosis, we highlight the lower IR in those patients on cyclosporin or tacrolimus compared to those without them. The scant exposure to other biologic agents hindered the evaluation of their respective IR. Finally, the IR in patients with and without antifibrotics did not differ.

**Table 3:** Incidence rate of functional deterioration by different variables

	Pacs-semester	Events	TM 100 pacs-semester	IC 95%
Hospital centers				
FJD	238.4	59	24.7	19.2–31.9
HCSC	150.1	36	23.9	17.3–33.2
HiLe	5.7	0	0	–
HIS	6.9	0	0	–
HPH	33.7	12	35.6	20.2–62.7
HUG	9.2	0	0	–
HULP	18.8	4	21.3	7.9–56.7
Gender				
Women	325.5	79	24.3	19.4–30.2
Men	137.3	32	23.3	16.4–32.9
Age in years				
≤ 60	156.9	34	21.6	15.5–30.3
60–69	164.0	43	26.2	19.4–35.3
70–79	93.4	26	27.8	18.9–40.9
≥ 80	48.5	8	16.5	8.2–32.9
Active-former smokers				
No	155.8	43	27.6	20.4–37.2
Yes	307.0	68	22.15	17.4–28.09
Comorbidity overall				
No	145.8	36	24.6	17.8–34.2
Yes	302.3	72	23.9	18.9–30.0
Comorbidity types				
CV disease				
No	225.5	49	21.7	16.4–28.7
Yes	222.6	59	26.5	20.5–34.2
History of cancer				
No	417.6	106	25.4	20.9–30.7
Yes	30.4	2	6.4	1.6–26.2
Liver disease				
No	410.6	100	24.3	20.00–29.6
Yes	37.5	8	21.3	10.6–42.6
COPD-Bronchiectasis				
Yes	395.6	104	26.3	21.7–31.8
No	50.4	3	5.9	1.9–18.44
Renal failure				
Yes	446.6	108	24.18	20.0–29.2
No	1.5	0	0	–
Hypothyroidism				
Yes	397.6	94	23.7	19.3–28.9
No	50.6	14	27.7	6.6–63.3
GER				
Yes	403.5	97	24.0	19.7–29.3
No	40.2	10	24.8	13.4–46.3
Radiological ILD pattern				
NSIP	236.7	45	19.0	14.1–25.4
UIP	153.5	50	32.2	24.6–42.9
Others	72.5	16	22.0	13.5–36.0
Emphysema (HRTC)(n = 54)				
No	273.5	67	24.5	19.3–31.1
Yes	68.1	32	17.6	10.0–31.0
Fibrosis (HRTC) (n = 54)				
No	189.5	40	21.1	15.5–28.8
Yes	134.5	37	27.5	19.9–37.9
FVC%				
< 80	155.9	39	25.0	18.3–34.2
> 80	306.9	72	23.4	18.6–29.5
DLCO (n = 68)				
< 60	138.7	38	27.3	19.8–37.6
> 60	237.8	62	26.1	20.3–33.4
Rheumatoid Factor (n = 65)				
Negative	310.4	82	26.4	21.3–32.8
Positive	97.9	22	22.5	14.8–34.1
ANA antibodies (n = 73)				
Negative	113.9	18	15.7	9.9–25.07
Positive	336.3	90	26.7	21.7–32.9
Ro antibodies (n = 72)				
Negative	313.12	85	27.1	21.9–33.6
Positive	99.1	16	16.1	9.8–26.3
Glucocorticoids during follow-up				
No	184.6	43	23.3	17.2–31.3
Yes	278.2	68	24.4	19.3–31.0
DMARDs during follow-up				
MTX-LEF-AM				
No	432.7	103	23.8	19.6–28.9
Yes	30.1	8	26.5	13.3–53.0
AZA-MMF				
No	321.2	71	22.1	17.5–27.8
Yes	141.6	40	28.2	20.7–38.5
Cyclosporin-Tacrolimus				
No	432.5	106	24.2	20.3–29.6
Yes	30.3	5	16.5	6.8–39.6
Biologic agents (RTX)				
No	429.0	102	23.7	19.5–28.8
Yes	33.8	9	26.6	13.8–51.1
Antifibrotic agents during follow-up				
No	439.1	106	23.9	19.7–28.9
Yes	23.76	6	25.2	11.3–56.1

Centers: Hospital Clínico San Carlos (HCSC), Hospital Infanta Leonor (HiLe), Hospital Infanta Sofía (HIS); Hospital Puerta de Hierro (HPH) Hospital Getafe (HUG), Hospital Universitario de la Paz (HULP), and Hospital Fundación Jiménez Díaz (FJD). ILD: Interstitial lung disease; UIP: usual interstitial pneumonia; NSIP: nonspecific interstitial pneumonia; others: indeterminate; lymphoid interstitial pneumonia, cryptogenic organizing pneumonia; alveolar pattern, mixed pattern; FVC%: predicted forced vital capacity; DLCO%: predicted diffusing capacity of the lungs for carbon monoxide; DMARDs: disease modifying antirheumatic drugs; methotrexate (MTX), leflunomide (LEF), antimalarials (AM); azathioprine (AZA), mycophenolate mofetil (MMF); RTX: rituximab. Antifibrotic agents: pirfenidone or nintedanib

Univariate analyses are detailed in Table 4. To examine the possible effect of therapeutic alternatives on slowing down pulmonary impairment a final model was developed (Table 5). It was adjusted for age, sex, year of diagnosis and baseline FVC%. Center did not influence and could be dropped from the model. We did not find statistical association between glucocorticoids, csDMARDs nor biologic agents. As expected, a UIP pattern was associated with a higher risk of pulmonary impairment compared to NSIP. Other variable independently associated with lower risk were the presence of Ro antibodies. Despite the number of events we tried to force the model including the use of antifibrotics, however the model did not vary (HR: 0.63 [0.32–1.28]; p = 0.21). The proportionality of these models was tested (*p* = 0.9).

**Table 4:** Bivariate analysis

	HR	IC 95%	p
Hospital centers			
FJD	1	–	–
HCSC	0.99	0.66–1.48	0.9
Others	0.91	0.46–1.08	0.78
HiLe			
HIS			
HPH			
HUG			
HULP			
Gender, male	0.92	0.62–1.35	0.6
Age in years			
≤ 60	1	–	–
60–69	1.18	0.77–1.80	0.4
70–79	1.29	0.72–2.3	0.28
≥ 80	0.75	0.41–1.38	0.36
Active-former smoker	0.77	0.52–1.11	0.2
Year of ILD diagnosis (5 year intervals)			
2007–11	1	–	–
2012–16	1.53	0.78–3.01	0.2
2017–20	2.54	1.1–5.54	0.018
Comorbidity (Yes vs No)			
CV disease	1.19	0.82–1.72	0.3
Non CV disease	0.76	0.48–1.19	0.23
ILD associated fibrosis (HRTC) (n =	1.13	0.75–1.70	0.5
ILD associated emphysema (HRTC)	0.7	0.35–1.37	0.3
ILD associated pulmonary (HRTC) hypertension	1.04	0.56–1.93	0.8
Radiological ILD pattern			
NSIP	1	–	–
UIP	1.75	1.18–2.6	0.005
Others	1.12	0.7–1.81	0.6
FVC% ≥ 80	0.94	0.64–1.37	0.7
DLCO% ≥ 60	0.91	0.63–1.32	0.6
ERS	1.0	0.99–1.01	0.5
Glucocorticoids	0.98	0.71–1.36	0.9
DMARDs (specific DMARD vs others)			
Conventional synthetic DMARDs			
MTX-LEF-AM	1.17	0.68–1.99	0.5
AZA-MMF	1.23	0.82–1.84	0.3
Cyclosporin-Tacrolimus	0.63	0.36–1.11	0.1
Biologic agents (RTX)	1.15	0.57–2.3	0.6
Antifibrotics agents	1.07	0.49–2.32	0.8
O_2_ during follow-up	1.39	0.96–2.02	0.07
Rheumatoid Factor + (≥ 40)	0.85	0.49–1.45	0.58
ANA antibodies + (≥ 1/320)	1.71	0.96–3.04	0.065
Ro antibodies +	0.59	0.39–0.88	0.01

*Centers* Hospital Clínico San Carlos (HCSC), Hospital Infanta Leonor (HiLe), Hospital Infanta Sofía (HIS); Hospital Puerta de Hierro (HPH) Hospital Getafe (HUG), Hospital Universitario de la Paz (HULP), and Hospital Fundación Jiménez Díaz (FJD). ILD: Interstitial lung disease; UIP: usual interstitial pneumonia; NSIP: nonspecific interstitial pneumonia; others: indeterminate; lymphoid interstitial pneumonia, cryptogenic organizing pneumonia; alveolar pattern, mixed pattern; FVC%: predicted forced vital capacity; DLCO%: predicted diffusing capacity of the lungs for carbon monoxide; DMARDs: disease modifying antirheumatic drugs; methotrexate (MTX), leflunomide (LEF), antimalarials (AM); azathioprine (AZA), mycophenolate mofetil (MMF); RTX: rituximab. Antifibrotic agents: pirfenidone or nintedanib. O2: oxigenotherapy

**Table 5:** Multivariate analysis

	HR	IC 95%	p	HR	IC 95%	p	HR	IC 95%	p
Gender, male	0.83	0.59–1.18	0.29	0.74	0.32–1.72	0.48	–	–	–
Age in years	1.00	0.98–1.02	0.9	–	–	–	1.04	1.01–1.07	0.011
Time of diagnosis by 5 year intervals	1.46	1.06–2.02	0.021	1.37	0.74–2.52	0.3	1.88	1.3–2.58	0.000
Radiological ILD pattern									
NSIP	1	–	–	–	–	–	–	–	–
UIP	1.75	1.27–2.39	0.001						
Others	0.93	0.48–1.82	0.8						
FVC% > 80	0.82	0.56–1.21	0.3	0.62	0.39–0.99	0.04	–	–	–
Glucocorticoids	0.94	0.62–1.41	0.7	1.07	0.59–1.93	0.8	0.53	0.34–0.83	0.006
Inmunomodulator therapy (csDMARDs; MTX, LEF, AZA, MMF, Tacro, AMs or biologic agents)				0.85	0.36–2.01	0.7	1.31	0.7–2.43	0.37
csDMARDs (MTX, LEF, AZA, MMF, Tacro, AMs)	1.13	0.75–1.71	0.5						
Biologic agents: RTX	0.91	0.41–2.05	0.8						
Ro antibodies +	0.61	0.39–0.95	0.032	–	–	–	0.36	0.19–0.65	0.001
ANA antibodies + (≥ 1/320)	–	–	–	3.76	1.88–7.53	0.000	–	–	–

Adjusted for age, sex, time calendar and disease severity at baseline (FVC%)

*ILD* interstitial lung disease, *UIP* usual interstitial pneumonia, *NSIP* non specific interstitial pneumonia, *others* indeterminate; lymphoid interstitial pneumonia, cryptogenic organizing pneumonia; alveolar pattern, mixed pattern; FVC%: predicted forced vital capacity; csDMARDs: conventional syntetic disease modifying antirheumatic drugs; methotrexate (MTX), leflunomide (LEF), antimalarials (AM); tacrolimus (TACRO) azathioprine (AZA), mycophenolate mofetil (MMF). RTX: rituximab

We stratified by radiographic patterns to perform an in-depth study. In NSIP subgroup, only two patients were on antifibrotics and none of them developed pulmonary impairment. In the final model, age and centers did not influence and dropped from the model. We group csDMARDs and biologic agents in a single variable due the small number of prevalence. Interestingly the presence of ANA antibodies ≥ 1/320 increased the risk of pulmonary impairment regardles other factors (Table 4). To see specific role of inmunomodulator therapy, this variable was changed in the final model for AZA-MMF versus the rest (HR: 1.23 [0.56–2.68], p = 0.60); or for calcineurin inhibitors versus the rest (HR: 0.43 [0.18–0.998], p = 0.05) or rituximab versus the rest (HR: 1.55 [0.71–3.38], p = 0.26). The proportionality of these models was tested (p = 0.8).

In the final model of UIP subroup (n = 29) neither gender, baseline FVC% nor centers influenced in the main outcome and were dropped. We had to group csDMARDs and biologic agents in one variable due the small prevalence. Interestingly, age and the presence of Ro antibodies, influenced in the main outcome. Other serologic parameters did not influence. Remarkably, glucocorticoids decreased the risk of functional impairment, but inmunomodulatory therapy (csDMARDs and biologic agents) did not achieve statistical signification (Table 4). We forced the model including the use of antifibrotics, however the model did not vary (HR: 1.18 [0.62–2.23]; p = 0.59). To see the specific role of inmunomodulator therapy on functional impairment, this variable was changed in the final model for AZA-MMF versus the rest (HR: 1.34 [0.81–2.22]; p = 0.2) with no variation of the rest of variables. Nevertheless, when immunomodulatory therapy was replaced for the variable rituximab versus the rest (HR: 0.41 [0.14–1.21], p = 0.1) glucocorticoids lost statistical significance (HR: 0.66 [0.42–1.04], p = 0.07). These are interesting data, although, the number of events were quite small to establish robust conclusions. The proportionality of these models was tested (p = 0.7).

## Discussion

The natural history of IPAF is still not well known IPAF is a heterogeneous entity in its own definition. Moreover, there are few retrospective studies, from a single center and with a short follow-up time. This study is the first observational multicenter study to date that has evaluated the effect of corticoids and immunomodulatory therapy on IPAF in terms of pulmonary functional impairment and in the long term. Moreover, we confirm previously identified risk factors of progression in IPAF, and propose new ones such as prognostic biomarkers.

As in other studies, IPAF NEREA patients were predominantly women, in their sixties, being NSIP the most frequent radiological pattern, and the most common clinical and serological manifestations were joint symptoms, Raynaud's phenomenon, and seropositive ANA antibodies [16, 26, 27].

In ILDs, a decline of pulmonary function is expected over time. Whereas DLCO% is highly sensitive for predicting the presence of ILD, lung volumes might be more useful for assessing disease extent [28]. Recent publications suggest that a 5% difference in FVC% is clinically meaningful in the short term [29]. Thereby, the current study has assessed the incidence rate of functional impairment as a decline in relative FVC% ≥ 5% over 6-month periods [21]. In our study, 63% of IPAF suffered deterioration over the study course, with an incidence rate of 23.9 per 100 patients-semester. Moreover, it is also important to note that our IPAF progressed during the first years of disease, as 50% of them developed functional deterioration at 16 months after IPAF diagnosis. To date, there are no studies that analyze long-term pulmonary functional progression, although as in other ILDs, this is a determining factor in the definition of a progressive phenotype with prognostic and therapeutic implications [30, 31]. Our results are similar to those shown in patients with rheumatoid arthritis-related ILD from the NEREA registry [32].

Another relevant matter we have addressed, is the identification of factors related to pulmonary functional impairment in IPAF. UIP pattern entailed a poorer prognosis regardless other factors, as it has been shown in patients with other CTD-ILD [32, 33]. Similarly, UIP pattern has been reported to be associated with an increased mortality risk both in IPAF [15, 16, 34, 35] and CTD-ILD [36, 37]. Regarding therapeutic management, we were not able to show differences among glucocorticoids nor inmunomodulatory therapy in the entire IPAF population. This could reflect the variability among patients and therapies. “It is important to note that therapeutic decisions in our patients were multidisciplinary, considering the patient health status, the extra-pulmonary disease, the ILD severity, and patient’s preferences in all cases.”

In order to study treatment effect in detail, we stratified the patients according to radiographic patterns and found that systemic glucocorticoids were associated with better outcome in UIP pattern. This points out the existence of different pathogenetic mechanisms in autoimmune-related UIP as compared to idiopathic forms [38]. This finding does occur in other CTD-ILDs [39], which have led to propose the use of glucocorticoids in progressive fibrosing phenotypes [19, 32]. Joerns et al. showed that the combination of glucocorticoids and MMF was associated with less functional progression after adjustment for radiological patterns [19]. In our study and regarding NSIP patients, it seemed that azathioprine and MMF didn´t influence, but with tacrolimus and cyclosporine, the statistical significance was reached. In the case of UIP pattern, a possible benefit of rituximab was found (p = 0.1). However, a small number of events supported our findings, and they need to be interpreted with caution.

In most studies, older age in IPAF can be considered a poor prognosis independent factor [34, 35, 40], although they were related to mortality. In our study we achieved statistical significance in those with UIP pattern, losing significance in the entire IPAF population and in those with NSIP pattern.

As a novelty, this study was able to evaluate the role of different antibodies on prognosis. Interestingly, the presence of Anti-Ro antibodies was independently associated with a lower risk of functional progression, both in NIU pattern and in the entire IPAF population. Whereas, the presence of ANA at titers ≥ 1/320 increased the risk of bad outcome in UIP subgroup. All these findings, need to be replicated in larger studies, in order to confirm their potential role as biomarkers in IPAF.

Taking the long period of inclusion into consideration, the analysis was adjusted for time calendar to elude bias. Intriguingly, a more recent diagnosis was associated study with greater functional progression, both in IPAF population and in UIP patterns. It is known that these patients are difficult to classify, mainly those with UIP pattern, which could have been previously diagnosed with idiopathic pulmonary fibrosis instead of IPAF, remaining outside this population in those earlier periods, but not in the most recent ones.

As major limitations of our study, is the retrospective nature of the design until 2017 and also the small sample size. Nevertheless, the inclusion of non-selected patients from seven hospitals with a long-term follow-up provides important strengths, giving an overall vision of real world evidence in this field. Moreover, all the patients were recruited and attended at special CTD-ILD multidisciplinary units, implicating standardized and homogeneous evaluations. All data were available for analysis, allowing adjustment for confounders to elude possible bias.

Our results support that patient with IPAF suffer pulmonary functional impairment that can be comparable to other ILD-CTD. The effect of certain treatments, as well as the identification of associated factors on pulmonary deterioration in patients with IPAF, may help clinicians in daily practice. Notwithstanding, our findings need to be replicated in additional prospective studies to gain further insight into the management of IPAF.

## Conclusions

Our results support that IPAF patients have relevant functional impairment over time that will impact their prognosis. Several factors predicting deterioration differ between radiographic patterns. This study was able to evaluate the role of different antibodies on prognosis, founding that the presence of Anti-Ro antibodies was independently associated with a lower risk of functional progression. The potential benefit of particular therapies in IPAF, as well as the identification of associated factors on progression, such as sociodemographic characteristics, radiological patterns, functional parameters, and autoantibodies is crucial for patient management.

## Data Availability

The datasets used and/or analysed during the current study are available from the corresponding author on reasonable request.
